# Characterizing bulk rigidity of rigid red blood cell populations in sickle-cell disease patients

**DOI:** 10.1038/s41598-021-86582-8

**Published:** 2021-04-12

**Authors:** Mario Gutierrez, Mark Shamoun, Katie Giger Seu, Tyler Tanski, Theodosia A. Kalfa, Omolola Eniola-Adefeso

**Affiliations:** 1 Department of Chemical Engineering, University of Michigan, Ann Arbor, MI 48109 USA; 2 Department of Pediatric Hematology/Oncology, University of Michigan, Ann Arbor, MI 48109 USA; 3 Cancer and Blood Disease Institute, Cincinnati Children’s Hospital Medical Center, University of Cincinnati College of Medicine, Cincinnati, OH USA; 4 Department of Biomedical Engineering, University of Michigan, Ann Arbor, MI 48109 USA; 5 Macromolecular Science and Engineering Program, University of Michigan, Ann Arbor, MI 48109 USA

**Keywords:** Biomedical engineering, Characterization and analytical techniques

## Abstract

In this work, we utilized a parameterization model of ektacytometry to quantify the bulk rigidity of the rigid red blood cell (RBC) population in sickle cell disease (SCD) patients. Current ektacytometry techniques implement laser diffraction viscometry to estimate the RBC deformability in a whole blood sample. However, the diffraction measurement is an average of all cells present in the measured sample. By coupling an existing parameterization model of ektacytometry to an artificially rigid RBC model, we formulated an innovative system for estimating the average rigidity of the rigid RBC population in SCD blood. We demonstrated that this method could more accurately determine the bulk stiffness of the rigid RBC populations. This information could potentially help develop the ektacytometry technique as a tool for assessing disease severity in SCD patients, offering novel insights into the disease pathology and treatment.

## Introduction

Sickle Cell Disease (SCD) is one of the most common and complex genetic blood disorders worldwide^1–3^. SCD originates as a mutation of the gene encoding for the oxygen-binding protein, hemoglobin. There are multiple genotypes of this hereditary blood disorder, all characterized by the production of mutated hemoglobin S (HbS)^3^. Red blood cells (RBCs) with a high content of HbS have a significantly reduced oxygen-binding capability and a high propensity to polymerize under hypoxic conditions^1,4^. The formation of a polymer nucleus and the increased concentration of unbound oxygen inside the RBC then induces oxidative damage on the cellular membrane, leading to a loss in membrane deformability^5^. The oxidative damage to the cell membrane also makes the RBCs prone to lyse, resulting in anemia and the release of hemoglobin into the open bloodstream, which damages the vascular wall, i.e., upregulation of inflammation markers^6–12^. As such, SCD is characterized by extremely detrimental symptoms, including pulmonary hypertension, anemia, and vaso-occlusion that can cause chronic organ damage^3^. These SCD symptoms are also accompanied by severe pain episodes known as “crisis” that represent a significantly reduced quality of life for SCD patients^3^. Chronic inflammation, vaso-occlusion, and overall negative symptoms in SCD originate from increased rigidity in the RBC membranes^3,11,13^.

The current understanding of SCD is focused mostly on the biochemical and genetic components of the disease. Conversely, the increased RBC membrane rigidity—separate from the sickled crescent-shape—is often overlooked as a significant factor in disease severity. Yet, the presence of stiff RBCs in blood was recently shown to reduce the vascular wall adhesion of white blood cells (WBCs) drastically and increase the vascular wall adhesion of platelet^14–16^. This works potentially hint at a more significant role for RBC rigidity in SCD patients’ well-being beyond instigating vaso-occlusion. The rigidity and hemoglobin composition of RBCs in SCD patients varies highly, and a high HbS content does not necessarily correlate to an increased cellular membrane stiffness^17^. For example, there can be a scenario with a large population of HbS concentrated RBCs (%S fraction) with moderate to low cellular membrane stiffness or another situation with a small %S fraction with RBCs with high membrane stiffness.

Thus, a full understanding of the impact of RBC rigidity in SCD symptom presentation would require the ability to characterize this property in individual patient blood. Therefore, several methods have been explored to characterize the mechanical properties of RBCs, including micropipette aspiration, atomic force microscopy, microfluidic devices, and optical tweezers. However, these methods are limited since they require single RBC isolation and are static techniques^18^. For one, the design and fabrication of robust microfluidic devices can be highly labor-intensive^19–21^. Conversely, traditional ektacytometry, or laser diffractometry, is more convenient and precise than other methods and thus has been adopted as a promising technique for testing RBC deformability^18^. Ektacytometry was initially developed and successfully used to screen for RBC membrane disorders such as hereditary spherocytosis^22^. This method in recent decades has been repurposed for the investigation of SCD^23^.

Ektacytometry principally works by inducing controlled shear stress on a suspension of RBCs in diluted blood. The resulting deformation from the applied shear stress is captured by shining a laser onto the sample, which creates a single diffraction pattern. The general technique can make three different types of measurements: (1) an Osmoscan which holds cells at constant shear stress while varying osmolality, (2) a deformability scan which keeps cells at a fixed osmolality while changing the shear stress, (3) an Oxygenscan which varies both the shear and oxygen gradient^24–26^. Cellular deformability, i.e., elongation, is then reported as an Elongation Index—a ratio of the difference between the major and minor axes in the cellular diffraction patterns over their sum. However, most works using ektacytometry to evaluate how RBC rigidity impact disease severity in SCD have focused on osmoscans^27,28^, which may not entirely recreate the in vivo setting. RBCs in blood flow are exposed to a relatively fixed in vivo osmolality (~ 290 mOsmol/kg) and varying shear as they transport through different blood vessels. Alternatively, measurements that employ analysis of RBC elongation in response to changes in shearing stresses at a fixed osmolality may offer a better description of RBC stiffness in SCD^23^. Oxygensans, the other form of shear-based analysis, are a robust method recently developed, which can measure the maximum RBC elongation at normal oxygen conditions and minimum RBC elongation under hypoxic conditions and point of sickling^26^. However, regardless of the shear-based method employed, ektacytometry is critically limited. It renders average deformability for all RBCs in the sample^23–25^, i.e., across both stiff and healthy RBCs. This average measurement of deformability may underestimate the rigidity of HbS-rich (%S fraction) or sickle RBC population in the blood, which could be a critical complication when it comes to assessing a patient’s well-being or treatment response. A sample with a small overall fraction of extremely rigid RBCs could render a similar elongation curve as a sample with a high fraction of moderately stiff RBCs. Previous studies have investigated how laser diffractions in ektacytometry differ based on non-homogeneous mixtures of RBC deformability^29^. However, to date, there is no established method for isolating and directly characterizing the rigidity of the HbS RBC population in patient blood.

Here, we present an approach to estimate the average rigidity of the HbS-rich (rigid) red cell population in SCD via a method that implements artificially rigidified RBCs mixed into whole blood at different rigid-to-healthy ratios. Said samples are measured via traditional shear-based ektacytometry deformability analysis. We then parameterize the measurements to develop a numerical model that predicts the rigid RBC population's bulk rigidity in SCD blood samples. This method can estimate the stiffness of sickled RBC populations with greater certainty, potentially serving as a platform for understanding disease severity in SCD patients and monitoring the efficacy of novel and established treatment options.

## Results

### Measurement of artificially rigid RBC populations in whole blood samples

**Figure 1:**
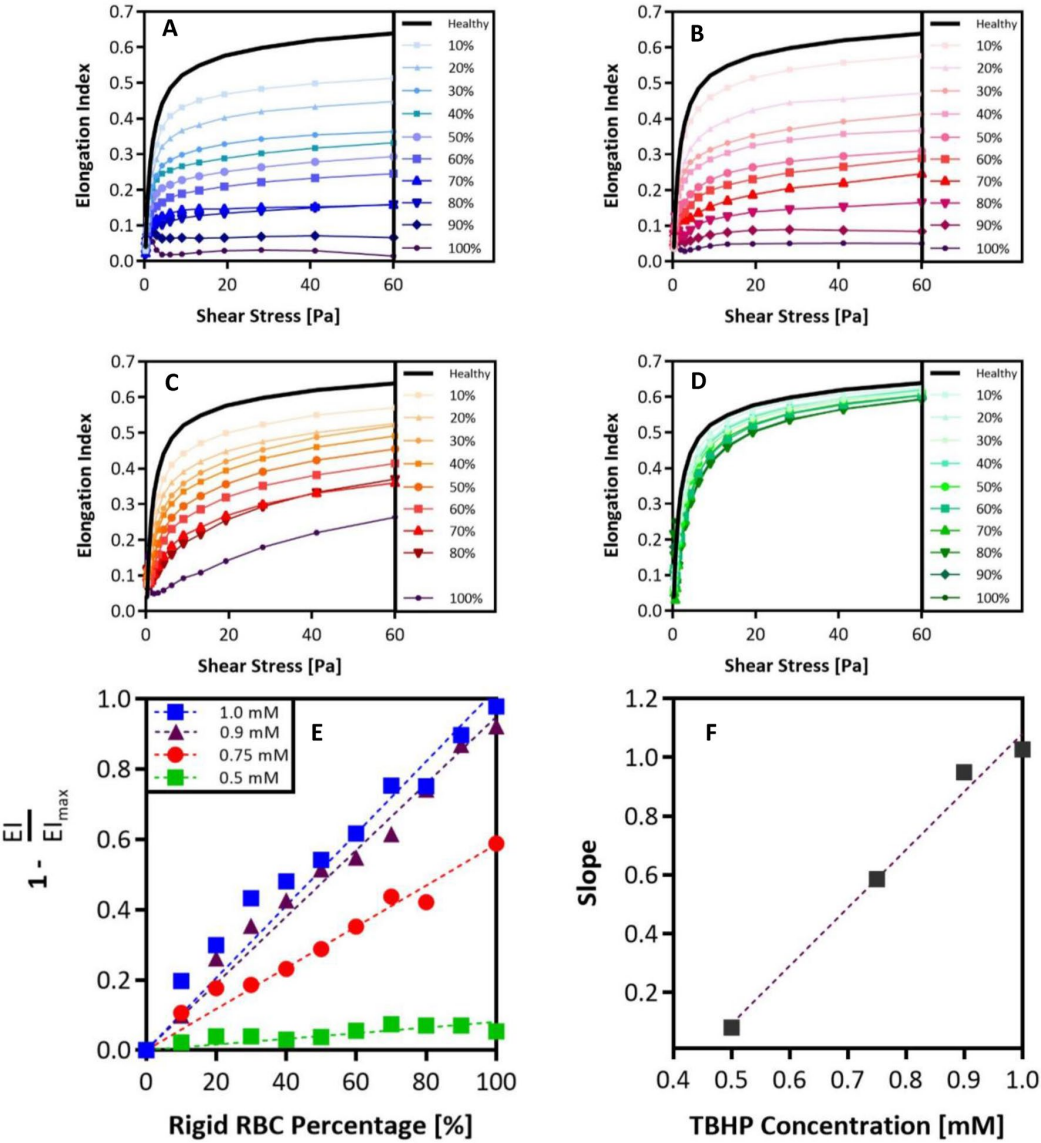
Ektacytometry analysis of various rigid RBC populations with varying RBC membrane stiffnesses. Ektacytometry curves for various rigid-to-healthy fractions ranging from 0 to 100% in increments of 10% rigid RBCs present. Rigid RBCs have been treated with: (**A**) 1.0 mM. (**B**) 0.9 mM. (**C**) 0.75 mM. (**D**) 0.5 mM TBHP. (**E**) Maximum EI recorded at 60 Pa for each rigid fraction condition is compared to EI_max_ of the healthy condition, these ratios are plotted as a function of rigid RBC fraction present. Linear regression is used to determine the slope of these rigid-to-healthy ratio trends. (**F**) Slopes of rigid-to-healthy ratio trends are plotted as a function of TBHP concentration.

In an ektacytometry scan, a collection of Elongation Index (EI) values plotted as a function of shear stress renders deformability curves. Previous work has determined that the critical factor in characterizing alterations in cell deformability is the maximum achievable EI, i.e., EI_max_^30^. Accordingly, measurements from healthy donors registered the highest EI_max_ values (~ 0.62). In contrast, SCD donors tended to have lower EI _max_ values. Next, we built an artificially rigid RBC approach, where the RBC membrane stiffness is easily altered to test how changing the fraction of rigid RBCs affects the EI_max_ of the whole blood. We artificially rigidified healthy donor RBCs via treatment with tert-butyl hydroperoxide (TBHP) in varying concentrations to obtain RBCs with a range of stiffness from *slightly* to *highly* rigid. Short interactions (< 60 min) with relatively low concentrations of TBHP (< 3.0 mM) induces lipid peroxidation and membrane protein crosslinking in RBCs. Prolonged exposure will result in structural alteration in protein networks and eventual RBC lysis^31^. We combined the stiffened RBCs with healthy (non-treated) RBCs in whole blood at different stiff-to-healthy ratios and measured the changes to the EI values^14^. The resulting deformability curves plotted along with the curve for the 100% healthy RBCs sample are shown in Fig. 1. As expected, ektacytometry measurements yielded smaller EIs as higher fractions of rigid RBCs were present in the blood. Thus, the samples with 100% treated RBCs registered the lowest EI_max_ for all TBHP concentrations evaluated. However, the spread between the curves became less pronounced with lower TBHP concentrations. Based on the 100% rigid fraction conditions, we observed that the *highly* stiff RBCs were the ones treated with 1.0 mM TBHP. The RBCs treated with 0.9 and 0.75 mM RBC treatments yielded *intermediate* stiffnesses while the 0.5 mM TBHP treatment yielded only *slightly* rigid RBCs.

### Building a predictive algorithm for estimating bulk stiffness of the rigid RBC population in sickle cell disease blood

To understand how the spectrum of rigid RBC fractions affects EI curves and EI_max_, we parameterized the curves in Fig. 1A–D. The EI_max_ recorded in every blood sample was divided by the average maximum EIs of healthy blood to produce a ratio of relative stiffness. Subsequently, we plotted these EI ratios as a function of rigid RBC fractions, as shown in Fig. 1E. We found a linear relationship between the EI_max_ achieved and the rigid RBC fractions in a blood sample. As expected, the *highly* rigid RBCs in blood had a more substantial impact on reducing the EI_max_ than *intermediate* and *slightly* rigid RBCs, as shown by the slopes of the linear trends in Fig. 1E plotted as a function of TBHP concentration in Fig. 1F. Parameterization of the relationship between the slope of rigid EI-to-healthy EI ratios versus TBHP concentration implies that we can interpolate between TBHP concentrations that were not experimentally tested. The correlations obtained by the parameterization of the EI measurements in Fig. 1 were imported into a MATLAB code to construct a predictive algorithm that can estimate the TBHP concentration, sample code available in supplementary material. Thus, the rigid RBC population's relative stiffness in a patient blood sample can be determined via two input variables: (1) EI_max_ recorded in the standard deformability measurement and (2) fraction of rigid or sickle RBCs in the patient’s blood sample.

Given the linearity of the relationships between the stiffness and fraction of the rigid RBC population in a blood sample, we hypothesized that our predictive algorithm could be used in combination with electrophoresis and hemoglobin analysis of patient blood to map the relative stiffness of the rigid (HbS) RBC populations in SCD. To this end, we measured the EIs for fifteen unique SCD patient blood samples. Table 1 lists the SCD genotype, age, gender, current medical intervention/therapies, %S fraction, and measured maximum bulk EI for all patient blood evaluated. A standard blood smear image was collected for patient 10, shown in Figure S1. The maximum EI of each measured patient blood sample (measured bulk EI_max_ in Table 1) combined with the corresponding %S fraction is used as initial inputs to estimate the *maximum* stiffness, i.e., EI_max_, of the HbS only RBC population for each patient. Meaning, we match each patient’s actual EI curve to an EI curve generated for an artificially rigid RBC blood that matches the measured bulk EI_max_ and %S (i.e., rigid RBC) fraction of the patient’s blood. The matched artificial EI curve, generated with the MATLAB algorithm, can then be backtracked to estimate what TBHP concentration can produce a curve that matches the actual raw patient deformability curve. Hence, the rigid RBC population’s apparent stiffness in the patient sample can be represented as a TBHP concentration. That is, the rigid RBC population in the SCD patient blood has a rigidity similar to the rigidity of healthy RBCs that had been artificially stiffened with a specified concentration of TBHP. Once we knew the representative TBHP concentration for a given patient blood sample, we extrapolated the % rigid fraction to a 100% to predict the maximum EI for 100% rigid RBCs, i.e., the maximum stiffness of only the rigid population or %S fraction.

**Table 1:** Sickle cell disease patient general information and ektacytometry maximum elongation values.

Patient	SCD Genotype	Age	Gender	Therapy	%S Fraction	Measured Bulk EI_max_	Predicted Rigid RBC Pop. EI_max_
#	-	Years	(M/F)	-	%	-	-
1	SS	13	M	CT	20.7	0.465	~ 0.01
2	SS	6	F	CT	48.7	0.421	0.198
3	SC	18	M	HU	50.3	0.465	0.299
4	SS	9	M	CT	75.2	0.266	0.145
5	SS	19	F	CT	21.1	0.539	0.188
6	SS	18	M	CT	34.4	0.462	0.136
7	SS	13	M	HU	89.9	0.297	0.259
8	SS	16	M	CT	43.1	0.401	0.095
9	SS	1.5	F	-	89.9	0.548	0.539
10	SS	15	M	HU	79.3	0.454	0.407
11	SC	17	M	HU	47.6	0.301	~ 0.01
12	SS	15	M	CT	55.8	0.482	0.362
13	SS	2	F	CT	37.4	0.606	0.561
14	SS	21	F	-	90.6	0.512	0.499
15	SS	15	M	HU	86.6	0.437	0.407

Information includes SCD genotype, age in years, gender (male or female), current patient therapy chronic transfusions (CT) or Hydroxyurea (HU), %S fraction as determined by blood electrophoresis, and maximum EI measured via ektacytometry in the actual SCD patient blood sample. The table also includes the predicted EI_max_ of rigid population, i.e., %S Fraction. Missing therapies indicate the patient is not under any type of treatment at the time of the measurement.

**Figure 2:**
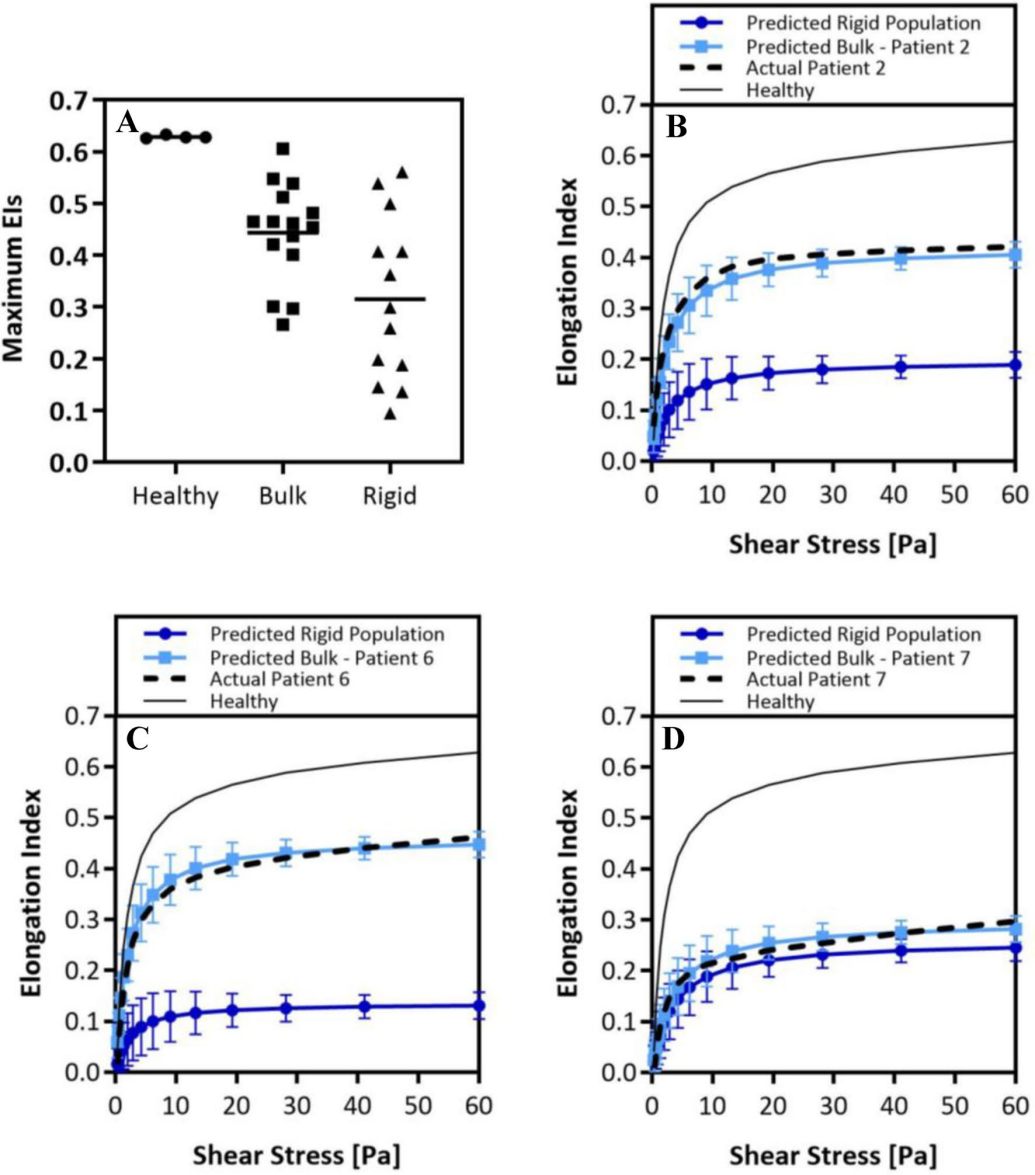
Predicting the rigidity of the rigid RBC populations in sickle-cell disease patients. (**A**), Average maximum EIs of *healthy* donors, actual *bulk* patient measured maximum EIs, and predicted maximum EIs of *rigid* population. Actual healthy and patient ektacytometry curves compared with predicted patient bulk ektacytometry curve and predicted curve of the rigid RBC population: (**B**), patient 2. (**C**), patient 6. (**D**), patient 7. Error analysis of the predicted EIs as a function of shear was performed using least square difference analysis. Error bars are plotted as standard deviation of the predicted elongation values. Student’s t-tests are performed to determine significance between Actual Patient curves and Predicted Bulk Curves, no significant difference is found for any patient using an α = 0.05, p-values > 0.05.

The values of the maximum EIs of measured patient bulk blood (bulk) are plotted in Fig. 2A next to maximum EIs obtained for healthy blood samples and the predicted maximum EIs of only the HbS (rigid) fraction for each patient’s blood. We observed a very narrow range in maximum EI of healthy blood measurements, while a wide range of maximum EIs is shown for SCD patient bulk blood measurements. Importantly, we saw a broader distribution in the maximum EI values of only the HbS populations with an average lower than the average maximum EIs of the raw SCD patient and healthy blood. From this data, we calculated ratios that compare the estimated maximum EIs of the sickle population in the patient blood samples to (1) the maximum EI in a healthy blood sample and (2) the maximum EI of the bulk SCD patient blood sample. A ratio value higher than unity suggests that the maximum EI of the rigid RBC population is much lower than what is measured by bulk blood measurement, thus underestimating the stiffness of the HbS population, i.e., sickled RBC, in an SCD patient.

1 $$Rigid\,to\,Healthy\,EI\,Fold\,Diff. = \frac{{Patient\,Predicted\,EI_{max}\,of\,Rigid\,Population}}{{Healthy\, EI_{max} \left( {\sim0.63} \right)}}$$

2 $$Rigid\, to \,Bulk\, EI \,Fold \,Diff. = \frac{{Patient\,Predicted\,EI_{max}\,of\,Rigid\,Population}}{{Patient\,Measured\,Bulk\,EI_{max}}}$$

3 $$EI\left( {SS} \right) = EI_{Max} \frac{{\left( {\frac{SS}{{SS_{1/2} }}} \right)^{m} }}{{\left( {\frac{SS}{{SS_{1/2} }}} \right)^{m} + 1}}$$

Subsequently, we used the predicted maximum EI of the rigid RBC population (shown as “rigid” in Fig. 2A) for each patient’s blood to create a deformability curve for the patient’s HbS only population, labeled as “Predicted Rigid Population” in Fig. 2B–D, according to an approach previously described by Baskurt et al^32^. This simplified Skreestra-Bronkhorst model, Eq. (1), also takes into account parameters, such as the shear stress associated with half the EI_max_ (SS_1/2_, also estimated in our parameterization model) and the range of shear stresses (SS) evaluated in the ektacytometry measurement. The variable “m” was a parameterization slope that was simply left at a value of 1. To confirm the validity of our approach to creating HbS, i.e., rigid population, only elongation curve, we again utilized the Skreestra-Bronkhorst plotting model, Eq. (3) to see if the deformability curves of the SCD patient bulk measurements can be replicated accurately, as well as a healthy deformability curve. We found an acceptable precision of the parameterization model when we compared the elongation curve derived from the actual measurement of a patient's bulk blood to the predicted representative curve for the patient’s blood. The precision is visually represented in Fig. 2B–D for patients 2, 6, and 7, which shows combined plots of the (1) predicted HbS only RBC elongation curve alongside the (2) predicted patient bulk deformability curve, (3) actual patient bulk deformability curve, and finally, the (4) a healthy deformability curve for reference. Supplementary Figure S2 shows similar plots for all patients evaluated in this study.

Interestingly, Figs. 2B and C show drastic differences in the *bulk* blood’s and the “sickle RBC” population’s deformability curves. In some cases, the rigid population yielded a maximum EI that was ~ 2 times smaller than what a bulk measurement yielded. However, in other cases, as shown in Fig. 2D, we observed very little difference between the actual raw bulk measurement and the predicted curve of the sickle.

## Discussion

One of the critical characteristics of SCD is the increased rigidity of the RBC membrane^3,11,13^. While there is evidence of its crucial role in the evolution of symptoms in SCD^33^, RBC rigidity is often disregarded as a minor side-effect of SCD rather than a critical biomarker. More than a mere inconvenience, RBC rigidity has the potential to be utilized as an indicator of a patient’s condition or response to new medications or therapies. For example, Gutierrez et al. showed that the presence of rigid RBCs in blood flow significantly reduced white blood cells (WBCs)’ adhesion to an inflamed vascular wall^14^. Interestingly, the results found a non-linear correlation between the fraction of rigid RBCs present in whole blood flow and the level of reduction in WBC adhesion across a range of RBC stiffness levels as explored in this work^14^. Additional work by Qiu et al. investigates the impact of rigid RBCs in blood flow on the functionality of the endothelium in a microvasculature-on-a-chip device^33,34^. Results from this work showed that even in the absence of vaso-occlusion and complications relating to hemolysis, endothelial dysfunction and increased permeability was detectable after only mechanical interactions with stiffened RBCs. Although these studies have begun to unravel the critical associations between RBCs stiffness and the complications seen in SCD, e.g., endothelial dysfunction and vaso-occlusion, there is still a need for further sophisticated models for investigating the effect of RBC stiffness in SCD. More intricate models also offer the potential to be used as analytical tests for novel therapeutics and understanding patient response to medication.

In this work, we presented an estimation method that can more precisely characterize the rigid RBC population’s stiffness in SCD based on the general shear-based ektacytometry measurement of patient blood. Originally developed as a screening tool for RBC membrane disorders^22^, and repurposed for the investigation of SCD, ektacytometry has yet to reached widespread clinical use. While ektacytometry has been proven to be a robust method for investigating RBC deformability, it is not perfect. The bulk blood measurement achieved via Ektacytometry deformability scans can register similar EI values for two different patients with vastly different stiffness in the rigid RBC populations due to variation in the HbS fraction population. Here, we implemented artificially rigid RBCs to evaluate rigid RBC fraction’s impact on bulk blood stiffness. Our results showed that ektacytometry deformability measurements are highly influenced by the degree of RBC rigidity in the sample and the fraction of rigid RBCs present (Fig. 1A–D). Samples containing rigid RBCs with a higher degree of stiffness showed the highest variability in deformability analysis upon change of the rigid fraction. These deformability measurements for artificially rigidified RBCs in blood allowed us to understand better how ektacytometry curves are altered by the amount of the stiff (i.e., sickled) RBC population in SCD blood. This understanding formed the base of our predictive model. Using the maximum EI measured and knowledge of the fraction of rigid RBCs in a mixed sample, we can estimate the rigid population’s stiffness in a patient’s blood.

The rigid conditions in SCD patient blood can vary depending on the medication, age, and genotype. We collected fifteen unique SCD whole blood samples from a diverse pool of donors ranging in SCD genotype, age, gender, and current therapy (Table 1). We determined the fraction of HbS RBCs, i.e., what we denote as the rigid population, in the patient sample via electrophoresis and HPLC hemoglobin analysis. Using what we learned from the artificially stiffened RBCs in the blood combined with a raw ektacytometry measurement of an SCD whole blood sample and hemoglobin data, we estimated the stiffness of the %S fraction, i.e., the rigid population in an SCD patient.

Our results showed that the rigid population’s stiffness in an SCD patient sample is often underestimated (Table 1). For patient 2, we see that the EI_max_ of the stiff RBCs, 48.7%, is 3.2 folds lower than the maximum EI given by the bulk raw measurement. Similarly, the EI_max_ of the stiff RBCs was 4.4, 4.7, and 6.7 folds smaller than the bulk blood value for patients 4, 6, and 8, respectively. This result implies the HbS RBCs, i.e., rigid population, in these SCD patients have a high degree of rigidity, which was similar to rigidity obtained for artificially rigidified RBCs incubated in 1.0 mM TBHP. Of note, the estimated stiffness of the rigid RBC population of patients 1 and 11 was predicted to be higher than the rigidity of our most stiff conditions measured and used to generate the numeric algorithm, i.e., RBCs treated with 1.0 mM TBHP. For these samples, the interpolative model cannot predict the rigid population’s max EI since it is out of range of our most stiff conditions measured (100% stiff fraction, 1.0 mM TBHP). Still, the max EI of the rigid population is many folds greater than the maximum EI in a healthy condition and the bulk raw standard measurement of the SCD sample. These conditions are denoted as > 10^∞^ under the *Rigid to Healthy EI Fold Difference* Column in Table 1. It is important to note that this analysis does not suggest that the maximum EI is always overestimated in every patient sample, but rather that it is a possibility. For example, for patients 9, 10, 13, 14, and 15, the maximum EI for the HbS RBCs was only ~ 1.1 times fold lower than the bulk raw measurement maximum EI. The estimated maximum EI of the rigid RBC population is compared to the maximum EI measured in the standard bulk deformability measurement, as shown in Fig. 2A. We observe that the average maximum EI for the rigid populations in the SCD patients is notably lower, ~ 0.32, compared to the standard bulk measurements, ~ 0.45. However, given the wide range of EIs constituting the averages, a statistically significant average was not calculated.

We did not observe any specific correlation between the current therapy, i.e., chronic transfusion or hydroxyurea, and the rigid population’s stiffness in an SCD patient sample. Hydroxyurea works by raising the levels of fetal hemoglobin, HbF, in the patient’s RBCs, which increases the oxygen binding capability and is expected to improve deformability in SCD RBCs^35–37^. However, one of the stiffest patient conditions we measured was that of patient 11, who was being treated with hydroxyurea at the time of the sample collection. One potential explanation for this discrepancy might be the shorter length of time the patient was on hydroxyurea medication than other patients on the same treatment. Also, we did not find a correlation between the stiffness of the rigid population and the patient genotype. Due to the availability of donors, thirteen of fifteen donors were genotype SS, and two donors were genotype SC. Again, one of the stiffest patient conditions measured was that of patient 11 with genotype SC. This observation is interesting since the genotype SC is typically regarded to be clinically less severe compared to genotype SS in regards to symptoms and occurrences of painful crises^3,38^.

For patients 2 and 6, we predicted the maximum EI of the rigid RBC populations to be 3.2 and 4.7 folds smaller than what was recorded in the measurement of their whole blood. That is, the predicted ektacytometry curves are shown to be considerably different from the bulk, raw measurements (Fig. 2B and C). On the contrary, we see there is not a considerable difference between the rigid population’s predicted curve and the actual bulk measurement in patient 7, with only a 1.2 times fold difference in maximum EIs, Fig. 2D. This visualization of the deformability curves shows our predictive model can estimate the rigid population’s stiffness in an SCD patient sample with greater precision.

Although the method presented here can predict with greater precision the stiffness of the rigid RBC population in an SCD patient blood sample, it is deficient in some areas. First, like traditional ektacytometry, our model cannot distinguish which RBCs have undergone irreversible shape change, i.e., configuration to a sickled crescent shape. Often a misunderstood disease, blood from SCD affected individuals is thought to be entirely composed of sickle-shaped RBCs, i.e., crescent-shaped^39^. However, the reality is that irreversibly sickle-shaped RBCs (ISCs) are highly fragile and short-lived^40^. Therefore, the majority of HbS-rich RBCs in circulation retain their discocyte-shape and yet remain rigid^40,41^. An example of this is shown in Supplementary Figure S1, wherein a standard blood smear of patient 10, genotype SS with a comparatively high %S fraction of 79.3%, only ~ 7.3% of RBCs in the smear show shape deformation from regular RBC discocyte shape. Second, others have reported that the presence of ISCs in a blood sample alters the elongation diffraction patterns imaged by ektacytometry^42^. That is, higher amounts of ISCs lead to more considerable differences in apparent bulk elongation indices, similar to results shown by Parrow et al. However, these studies focused on using Osmoscan analysis^23,42^.

Thirdly, our predictive model was built using the deformability measurements of artificially stiffened RBCs; Thus, the degree of stiffness in this model system was uniform regardless of %rigid fraction. This assumption is carried over when using the model to predict the stiffness of the %S fraction in an SCD patient blood sample. However, it is essential to note that there is likely a range of stiffnesses in a patient’s %S RBC population, i.e., not all rigid RBCs have the same degree of rigidity. The novelty of our model is the capability to neglect the contribution of the healthy, i.e., HbA, RBCs in a standard ektacytometry measurement. Another point of interest is that the measurements performed in this study were standard deformability elongation analysis without any alteration in the blood’s oxygen conditions. A recent development in ektacytometry technology is the Oxygenscan feature^26^, which determines the point of sickling of HbS-rich cells in a blood sample by alternating oxygen conditions. However, in terms of measuring a maximum elongation index at maximum shear stress, the end-result is still the same regardless of oxygen conditions. Given our model is built using only information from maximum elongation, our approach is complementary to the Oxygenscan.

Finally, improvements to the ektacytometry method, as demonstrated here, could increase its prevalence as a reliable tool for measuring RBC rigidity. This approach can also lead to the utilization of ektacytometry as a tool for understanding patient response to therapies. For example, blood transfusions are a standard treatment for SCD patients^43^. A previous work has proposed reducing the %S population to less than 30% is beneficial for moderating symptoms in SCD patients^44^. However, the reasons why this is the case remain unexplored, and it is unclear what role the stiffness of the %S population plays in altering symptoms. ^44^ The prior work by Gutierrez et al. showing the presence of artificially rigid RBCs in blood flow reduces WBC adhesion to inflamed endothelium drastically, depending on the RBC rigidity level and fraction in blood^14^. Given the wide variability in the stiffness of HbS-rich RBCs from patient to patient demonstrated in Fig. 2A, the arbitrary prescription of reducing the %S population to less than 30% with transfusion may not be ideal for every patient. Specifically, some patients could have increase infection risk, depending on the amount and stiffness level of the HbS-rich RBCs present in the blood. Thus, the insight into the stiffness of the HbS-rich RBC populations offered by our presented method may be useful for optimizing transfusion therapies. In another example, as previously mentioned, Qiu et al. show that rigid RBCs’ mechanical impact alone was sufficient to create endothelial injury^33^. This damage to the endothelium led to inflammation, which causes endothelial dysfunction in SCD, contributing to crises^33^. Given that stiffened, HBS-rich RBCs have a higher propensity to marginate, i.e., migrate near the blood vessel wall^45^ ,we anticipate these altered cells are likely well-positioned to cause endothelial damage regardless of their composition in blood. Thus, understanding the rigidity level of the HbS population can offer insight into crisis risk. However, more clinical studies are necessary to probe this possibility thoroughly.

Overall, this work presents an innovative approach for a more thorough examination of the ektacytometry-based assessment of RBC stiffness in SCD patients. We utilize information from an RBC deformability measurement combined with standard electrophoresis and hemoglobin analysis to build a parameterization model to predict the rigid RBC population’s bulk rigidity in a patient’s blood with greater precision. Although extensive future work is needed to prove ektacytometry as a robust clinical tool, knowledge from the presented model offers a better understanding of the bulk stiffness in a rigid or sickle population of a patient blood sample. This information could help diagnose disease severity in SCD, understand the variation of infection susceptibility in different SCD patients, and assess how patients respond to blood transfusions or novel medications.

## Materials and methods

### Human study approvals

Informed Consent was obtained before blood collection from all subjects and parents of minors involved in the current study. The study received approval from the University of Michigan Internal Review Board (IRB-MED and IRB-HSBS). All procedures were conducted following the tenets of the Declaration of Helsinki.

### Preparation of SCD patient blood

Fresh blood was obtained on the day of a patient's routine clinical visit via venipuncture. Blood was drawn into standard Vacutainer Lavender K2-EDTA tubes (BD) and stored at 4 ºC. A single tube was taken from SCD patients. Blood samples were shipped in 4 ºC cold packs overnight to Erythrocyte Diagnostics Laboratory of the Cancer and Blood Diseases Institute at the Cincinnati Children’s Hospital for examination the following day. All SCD patient samples were measured within 24 h of blood draw.

### Preparation of human Non-SCD blood

Fresh blood was obtained from healthy, i.e., no SCD, donors on the day of ektacytometer measurements via venipuncture. Blood was drawn into standard Vacutainer Lavender K2-EDTA tubes (BD). Multiple tubes were taken from healthy donors. Blood from healthy donors was then separated from WBC-rich plasma via a series of slow-speed centrifugation steps. RBCs from healthy donors were washed thoroughly via suspension in phosphate-buffered saline (PBS -/-) and centrifugation. RBCs and WBC-rich plasma were stored at 4 ºC until artificial rigidification and reconstitution, which was done right before ektacytometry analysis.

### Red blood cell rigidification and sample preparation

Washed RBCs from healthy donors were suspended in a 2% hematocrit and incubated with a specific concentration of Luperox tert-butyl hydroperoxide (TBHP) (Sigma-Aldrich). Four parent concentrations of TBHP were chosen as base RBC rigidities: 1.0, 0.9, 0.75, and 0.5 mM TBHP. Incubation for 30 min induced the loss of RBC membrane flexibility. After adequate washing, stiffened RBCs were mixed with healthy RBCs in whole blood in increments of 10% rigid RBC fractions up to a total of 100% while holding the hematocrit constant at ~ 40%. No RBC lysis was detected for the 30-min incubation period for any of the TBHP concentrations evaluated.

### Ektacytometry deformability measurements

All samples, both healthy and SCD donors, were measured independently using a LoRRca Maxsis Ektacytometer (Mechatronics Instruments BV, Zwaag, The Netherlands) at the Erythrocyte Diagnostics Laboratory of the Cancer and Blood Diseases Institute at the Cincinnati Children’s Hospital. SCD whole blood samples and reconstituted healthy donor samples were diluted ~ 200 × in polyvinylpyrrolidone (MW 360,000) solution. The solution is then transferred into LoRRca automized measuring vessel. Measurements are taken through a range of shear stresses (Pa) up to a maximum of 60 Pa. Cell deformation is expressed as an elongation index calculated by Eq. (4), where A represents the major axis and B the minor axis in deformation. Elongation Index (EI) values are plotted versus shear stress (Pa) to obtain the deformability curve.

4 $$EI = \frac{A - B}{{A + B}}$$

### SCD patient blood analysis and characterization

Complete blood counts were run on a Sysmex 9100 XN automated machine. The phlebotomy team collected whole blood (~ 3 mL) into an EDTA lavender top tube (BD). The sample was then sent to the hematology lab at Michigan Medicine and run according to university protocol and manufacturer’s instructions. Results were verified and reported through the electronic medical records at the University of Michigan. Hemoglobin evaluation was run on a Bio-Rad Variant II cation exchange HPLC system by high-pressure liquid chromatography (HPLC). Whole blood (~ 3 mL) was similarly collected by the phlebotomy team in an EDTA lavender top tube and run per protocol. HPLC works by separating components of blood through interactions with the absorbent particles. Reports typically consist of five different hemoglobin genotypes, including Hgb S, Hgb A1, Hgb A2, Hgb C, and Hgb F.

## Supplementary Information

Supplementary Information
