# Supplementary material for: Characterizing bulk rigidity of rigid red blood cell populations in sickle-cell disease patients
Source: Sci Rep. 2021 Apr 12;11:7909. doi: 10.1038/s41598-021-86582-8 (PMC8041827; doi:10.1038/s41598-021-86582-8)
Supplement: Supplementary file 1 — Supplementary Information [file 41598_2021_86582_MOESM1_ESM.docx]

Characterizing bulk rigidity of rigid red blood cell populations in sickle-cell disease patients

**Supplementary Information**

Mario Gutierrez,^1^ Mark Shamoun,^2^ Katie Giger Seu,^3^ Tyler Tanski,^1^ Theodosia A. Kalfa,^3^ Omolola Eniola-Adefeso^1,4,5,*^

^1^Department of Chemical Engineering, University of Michigan, Ann Arbor, MI 48109.

^2^ Department of Pediatric Hematology/Oncology, University of Michigan, Ann Arbor MI, 48109

^3^Cancer and Blood Disease Institute, Cincinnati Children’s Hospital Medical Center, University of Cincinnati College of Medicine, Cincinnati, OH, United States

^4^Department of Biomedical Engineering, University of Michigan, Ann Arbor, MI 48109.

^5^Macromolecular Science and Engineering Program, University of Michigan, Ann Arbor, MI 48109.

**Supplementary Methods**:

The approximations shown in Table 1 and in Figure 2 are a result of the parameterization of the data shown in Figure 1. A sample code is shown in the supplementary material that show how the approximations build from the parameterization made in Figure 1. The code is organized into sections that are referenced in the description below.

- Essentially, a linear regression is performed on Figure 1F, where *y=mx+b,* y = slope of rigidity %fraction trend, m=slope of TBHP concentration trend, x=TBHP concentration, b=intercept of rigidity %fraction trend. This regression is done in excel, the slope and intercept values are fed into MATLAB code, Section 1 & 2.
- These regressions are then used to interpolate between TBHP concentrations and in turn, estimate how a TBHP concentration might result in a %fraction trend, such as the ones seen in Figure 1E. This is done in MATLAB code Section 3.
- Subsequently, using a rigidity %fraction trend, we then can approximate what the EI value might be at a given Rigid RBC percentage, including 100%, which would essentially be analogous to a rigid population. A sample calculation is done for Patient 6, MATLAB Section 6.

With this in mind, we then take data from raw ektacytometry measurements done on SCD patient samples. We take the maximum EI from this measurement along with approximating the %S fraction as the rigid fraction %, and using the parametrized trends mentioned above (Figure 1E & 1F), we approximate how stiff that rigid population might be.

- A sample calculation is done for Patient 6, MATLAB Section 6, all others in Section 8.
- With this information, we can extrapolate and predict the maximum EI of that rigid population and make comparisons to an EI_max_ of a healthy condition as well as the EI_max_ of the bulk measurement, these comparisons are done as fold difference ratios as described in Equation 1 & 2 and shown in Table 1. The EI_max_ values are plotted in Figure 2A. MATLAB Section 8.
- Leveraging the plotting model from Equation 3, we then take the estimated EI_max_ of the rigid population to predict what the ektacytometry curve of the rigid population might look like in comparison to the bulk measurement and a healthy blood measurement. This is shown for Patients 2, 6, and 7 in Figure 2, and for all other patients in the Supplementary Figures. MATLAB Section 9.

**Sample MATLAB Code**:

%Ektacytometry Parameterization - SCD Patient Curves

clc

close all

EI=0.633; %Maximum EI of a healthy curve%

p=1; %m power of (S/S_half, Skreestra-Bronkhorst plotting model equation (3)%

%%%%%%%%%%%%%% Section 0 - Importing the Raw SCD Patient Data from Excel %%%%%%%%%%%%%%

filename = 'Ekta_SCD';

sheet = 3;

xlRange_SCD = 'B6:Q20';

EIMaxRange = 'B22:Q22';

fracRange = 'B23:Q23';

shearsRange = 'A6:A20';

patientEIs = xlsread(filename,sheet,xlRange_SCD); %Matrix of Patient raw measured EIs

shears = xlsread(filename,sheet,shearsRange); %Utilized Shear Range

S = shears'; %Transposing shear vector

Es = xlsread(filename,sheet,EIMaxRange); %Vector of Patient Max EIs

fractions = xlsread(filename,sheet,fracRange); %Patient %S Fractions

frac = fractions/100; %Converting fractions from percent to decimal

%%%%%%%%%%%%% Section 1 - Linear Regression Model - Estimating EImax %%%%%%%%%%%%%%%%%%

S_slope=1.973; % Slope of the SLOPES in linear regression model, Slope of Figure 1F, Note that intercepts in Figure 1E were forced to zero%

I_slope=-0.8932; % Intercept of the SLOPES in linear regression model, Slope of Figure 1F%

%%%%%%%%%%%%% Section 2 - Linear Regression Model - Estimating S_half %%%%%%%%%%%%%%%%%

S_slo=-6.5587; % Slope of the SLOPES in linear regression model%

I_slo=5.8334; % Intercept of the SLOPES in linear regression model%

S_int=0.4576; % Slope of the INTERCEPT in linear regression model%

I_int=0.6226; % Intercept of the INTERCEPT in linear regression model%

S_half=2.35; %Constant Value - Guessed - Literature

S_half_heal=1.869182;% 1.8915; %Constant Value - Estimated - Average of Healthy SS0.5

%%%%%%% Section 3 - Output the [TBHP] needed to achieve such Emax and %Sfraction %%%%%%%

TBHP_need=((1-Es./EI)-(frac.*I_slope))./((frac.*S_slope));

TBHP_need(1,1)=((-1)*(I_slope/S_slope)); %Predicting TBHP concentration that does no longer affects RBCs in 30 min incubation

TBHP_out=round(TBHP_need,2);

Predicted_TBHPs=TBHP_out'

%%%%%%%%%%%%%%%% Section 4 - Estimating S_half for patient %S fractions %%%%%%%%%%%%%%%%

Y_slo = (S_slo)*(TBHP_need)+I_slo; %For S_half Prediction

Y_int = (S_int)*(TBHP_need)+I_int; %For S_half Prediction

for i = 1:length(frac)

for x = 1:length(Es)

J(i,x) = Y_slo(x)*frac(i)+Y_int(x);

end

end

Sh=diag(J)*S_half_heal; %Predicted S_half for all the patients

Es(1,1)=EI; %Replace the 0% value for the max EI in healthy

%%%%%% Section 5 - Creating matrix of shear stresses and EImax's calculated in Es %%%%%

E=zeros(length(S),length(frac));

for m = 1:length(S)

for n = 1:length(frac)

E(m,n)=Es(n)*(((S(m)/Sh(n))^p)/(((S(m)/Sh(n))^p)+1));

end

end

%%%%%%%%%% Section 6 - Predicting the 100% rigid for Patient 6 %%%%%%%%%%%

Yx=(S_slope).*(TBHP_need(1,7))+(I_slope);

EIx=EI*(1-Yx);

Y_slox = (S_slo)*(TBHP_need(1,7))+I_slo;

Y_intx = (S_int)*(TBHP_need(1,7))+I_int;

Shx=(Y_slox*1+Y_intx)*S_half_heal;

Ex=zeros(length(S),1);

for q = 1:length(S)

Ex(q)=EIx*(((S(q)/Shx)^p)/(((S(q)/Shx)^p)+1));

end

show=Ex %Predicted Rigid Population EI range as function of Shear stress range

%%%%%%% Section 7 - Error Estimation - Difference from Actual Curve & Predicted %%%%%%%

Dif=minus(patientEIs,E); %Finding the differences between the actual EI values and predicted

SqDif=Dif.^2; %Squaring the differences

SS=sum(SqDif,2); %Sum of the differences in each shear across patients

Var=SS/(length(S)-1); %Variance in each shear

StDevs=sqrt(Var); %Standard Deviation for each shear

Err100=StDevs(15,1); %Error at the maximum shear where EImax occurs

%%%%%%%%%%%%%% Section 8 - Estimating the EI max for the 100% Condition %%%%%%%%%%%%%%%%

stepsize=0.05; %Arbitrary step size for fractions from 0-100%

F=[0:stepsize:1]'; %Fraction vector

P=zeros(length(F),length(Yp3));

for z = 1:length(F)

for u = 1:length(Yp3)

P(z,u)=EI/(EI*(1-Yp3(u)*F(z)));

end

end

Pub=P(:,1:7);

Eh=EI*(1-Ys); %EImaxs at the 100% rigid %Sfraction for each patient, note fraction=1

Eh(1,1)=EI; %Replacing predicted healthy value with actual healthy value

healratio = round(((EI./Eh)'),2) %Ratio of Healthy EI (0.633) compared to EImax @100% rigid population

bulkratio = round(((Es./Eh)'),2) %Ratio of bulk max "raw" patient reading compared to EImax @100% rigid population

PHR=round(((EI-Eh)./EI)*100,0)' %How much smaller is the EI compared to Healthy in Percent

PBR=round(((Es-Eh)./Es)*100,0)' %How much smaller is the EI compared to Bulk in Percent

%%%%%%%%%%%%%%%%% Section 9 - Plotting Predicted and Actual Curves %%%%%%%%%%%%%%%%%%%%

t0='EI Curves for X'; %Title of plot

figure(1)

plot(S,patientEIs(:,1),'o-','LineWidth',2)

hold on

errorbar(S,E(:,1),StDevs,'k','LineWidth',1)

**Supplementary Figures**:

**Figure S1. Blood smear image of SCD patient 10**. Standard blood smear image of SCD patient 10, genotype SS. Patient 10, 15-year-old male on hydroxyurea therapy. Patient 10 has a %S fraction of 79.3% as determined by standard electrophoresis analysis. Only ~7.3% of RBCs in visible smear show shape deformation from regular RBC discocyte shape.


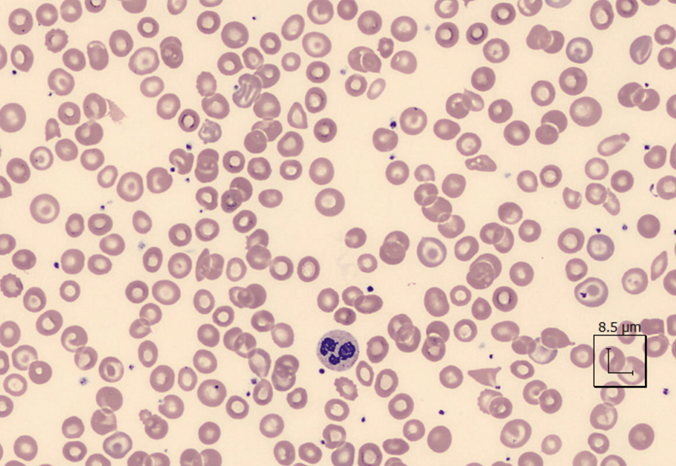


**Figure S2. Predicting the rigidity of the rigid RBC populations in sickle-cell disease patients**. Actual healthy and patient ektacytometry curves compared with predicted patient bulk ektacytometry curve and predicted curve of the rigid RBC population: **A**, patient 1. **B**, patient 3. **C**, patient 4. **D**, patient 5. **E**, patient 8. **F**, patient 9. **G**, patient 10. **H**, patient 11. **I**, patient 12. **J**, patient 13. **K**, patient 14. **L**, patient 15. Error analysis of the predicted EIs as a function of shear was performed using least square difference analysis. Error bars are plotted as standard deviation of the predicted elongation values. Student’s t-tests are performed to determine significance between Actual Patient curves and Predicted Bulk Curves, no significant difference is found for any patient using an α=0.05, p-values > 0.05.

**I**


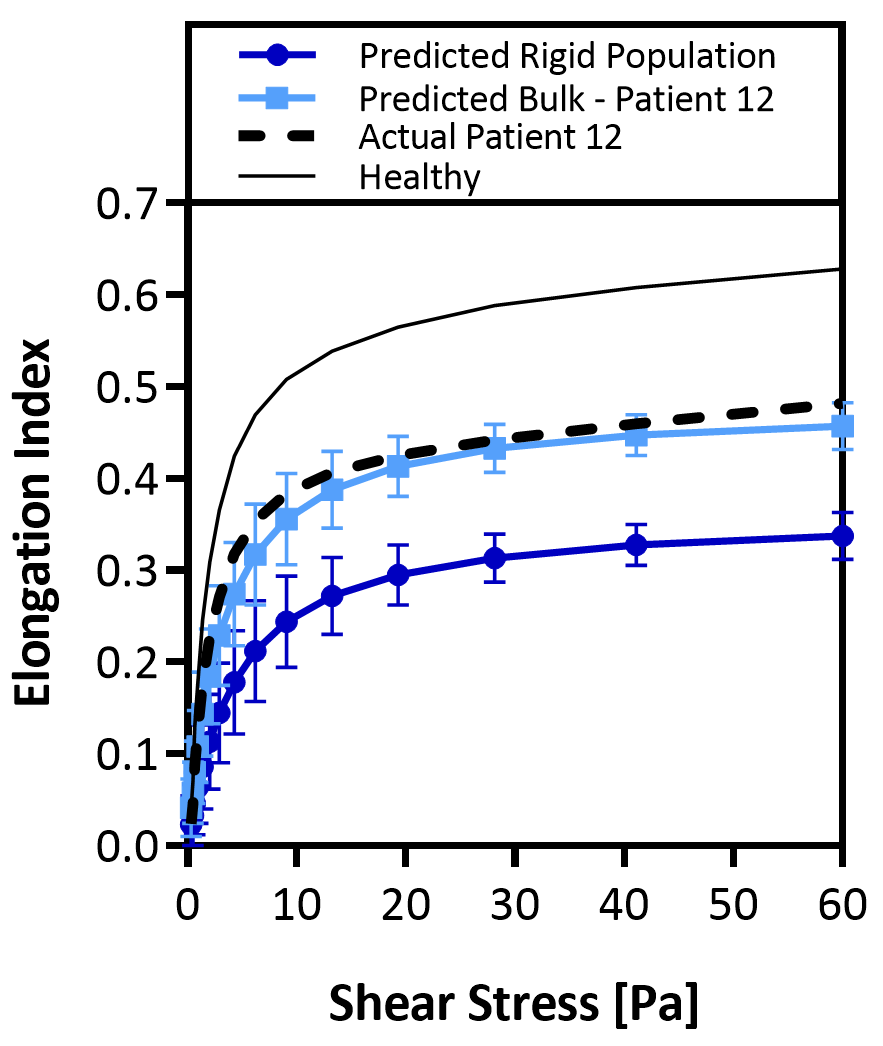


**H**


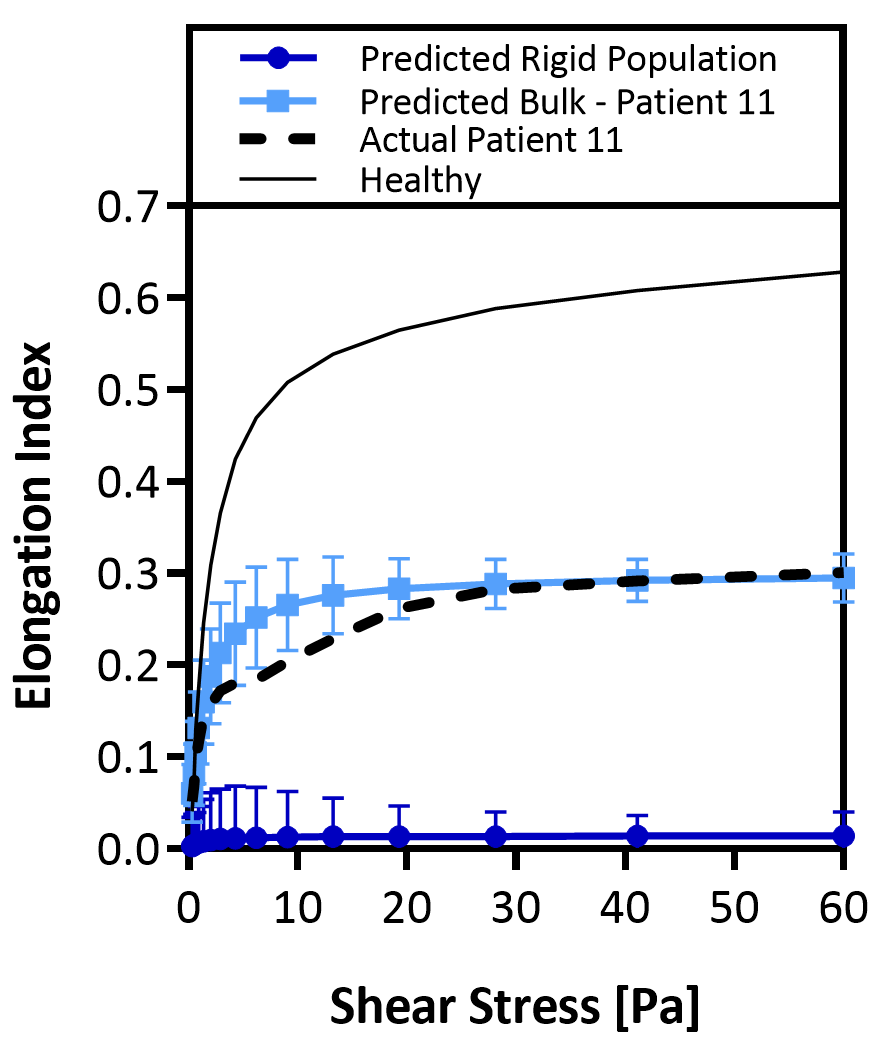


**G**


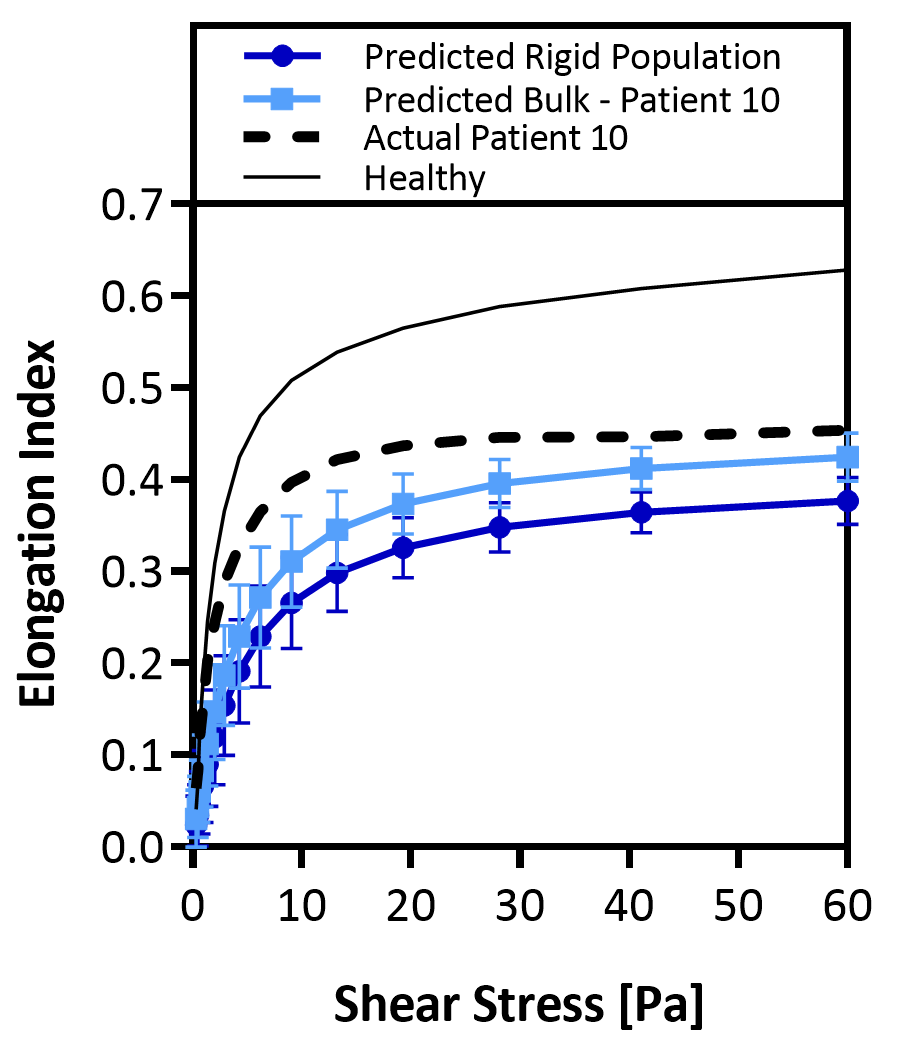


**F**


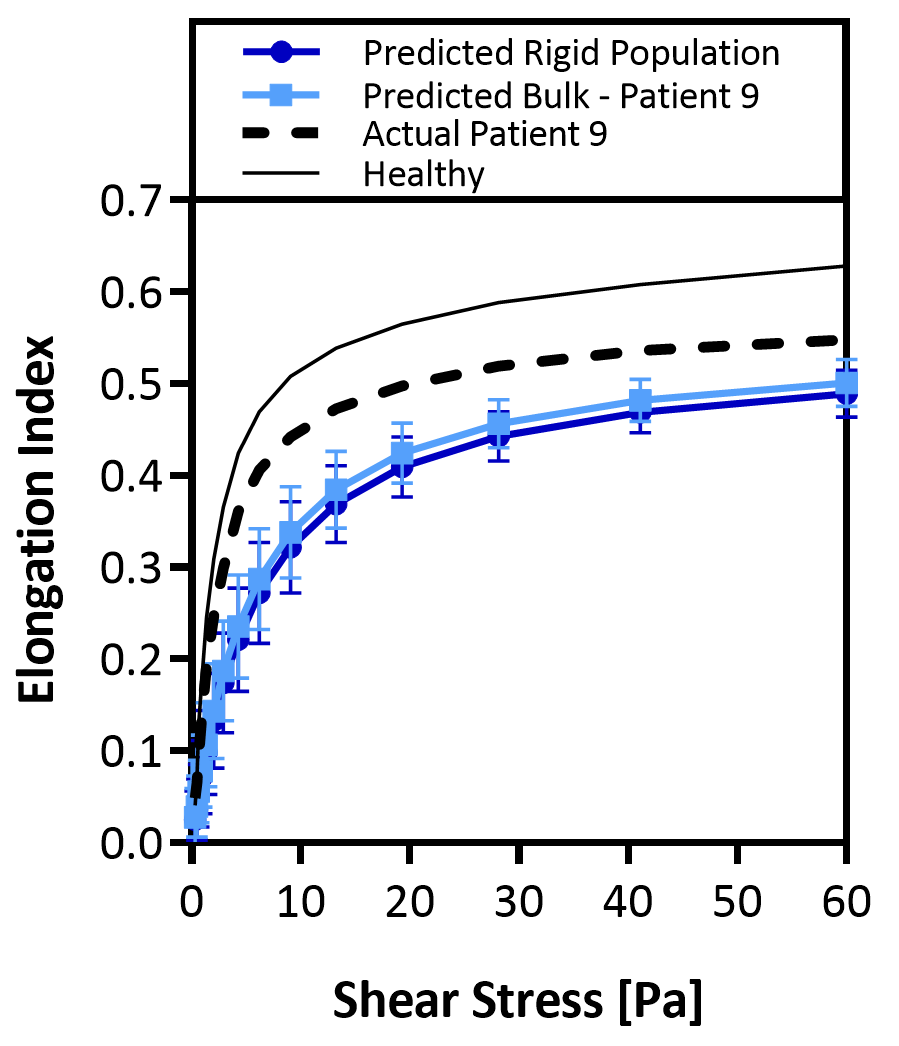


**E**


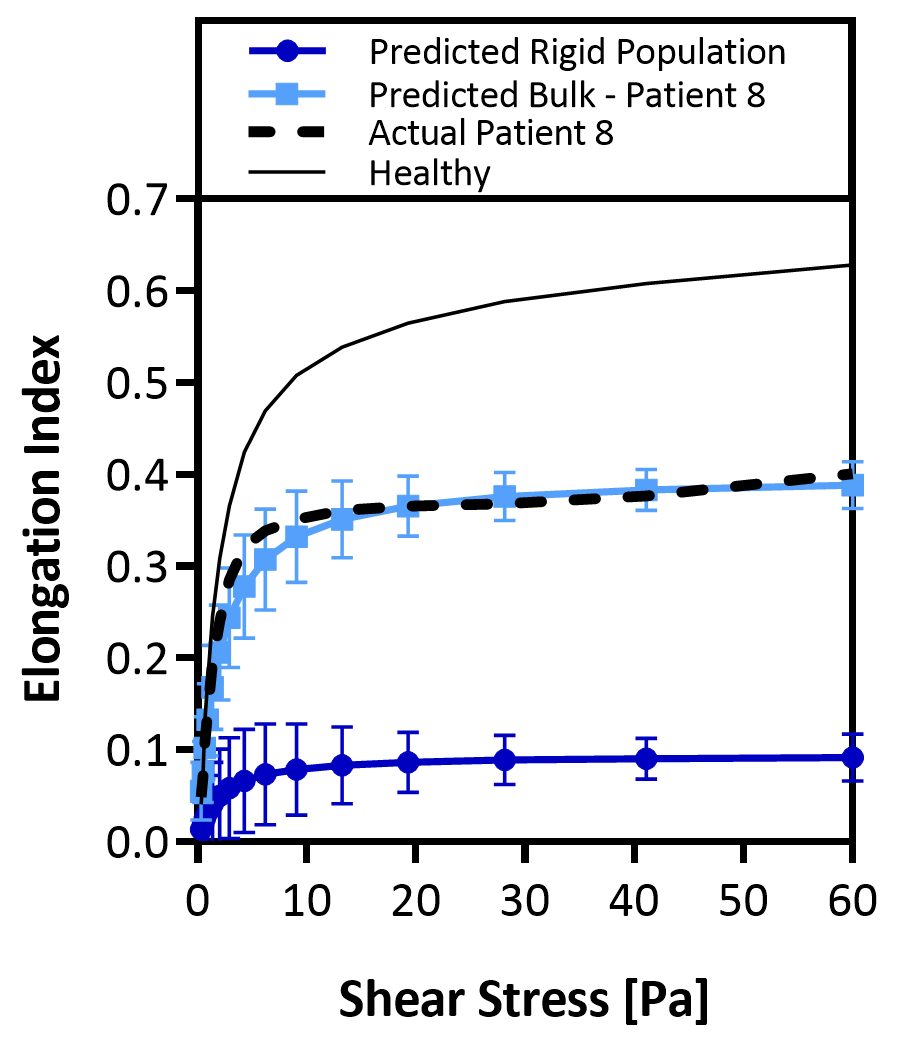


**A**


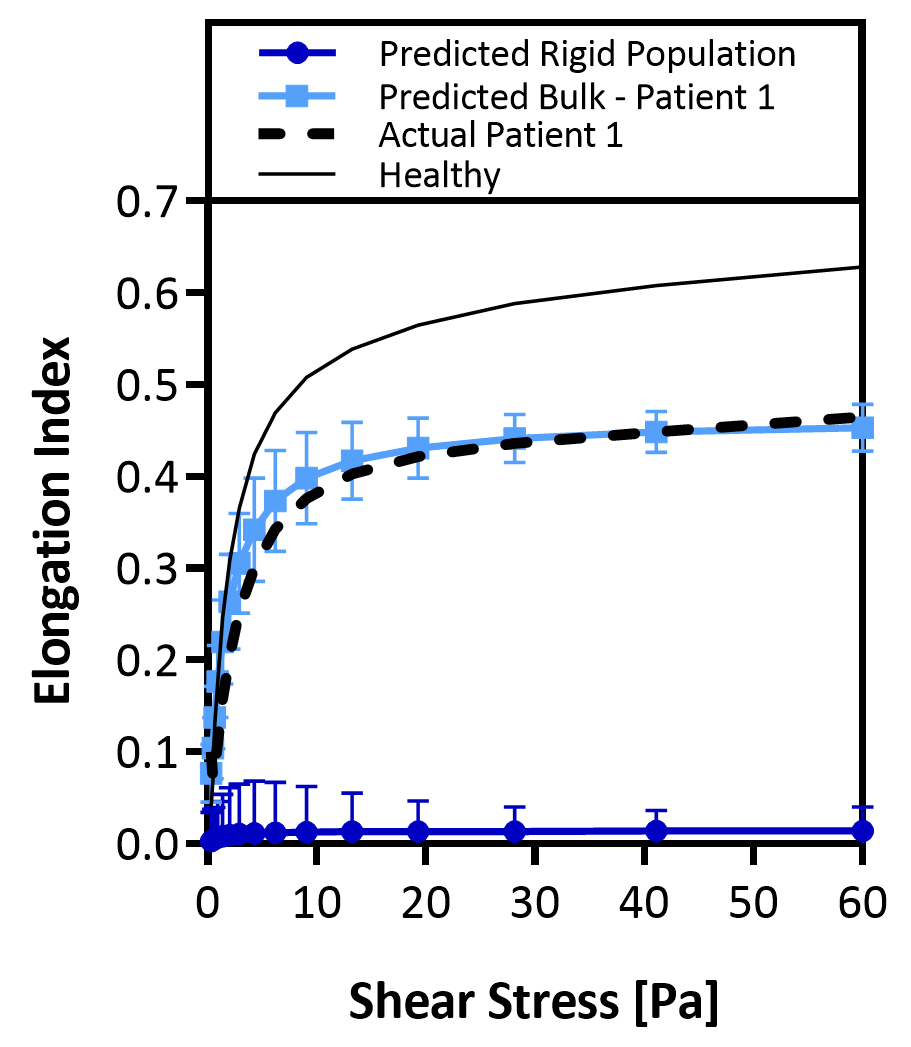


**D**


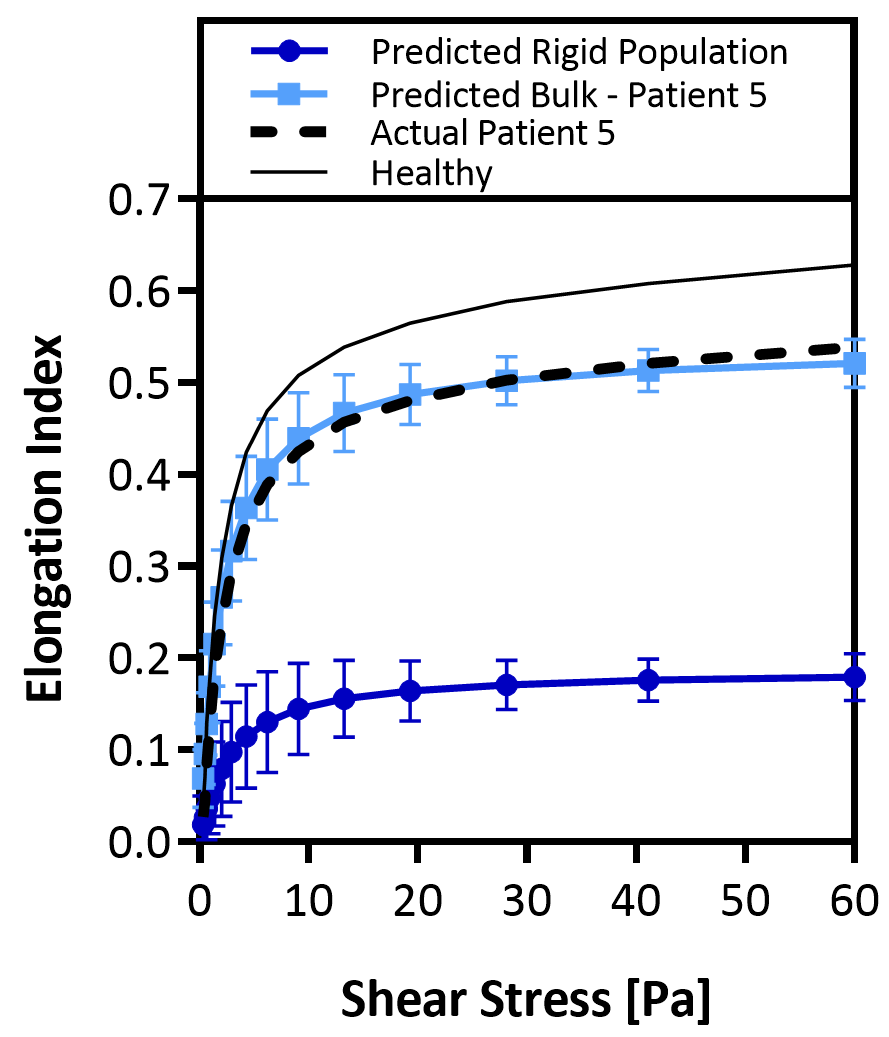


**C**


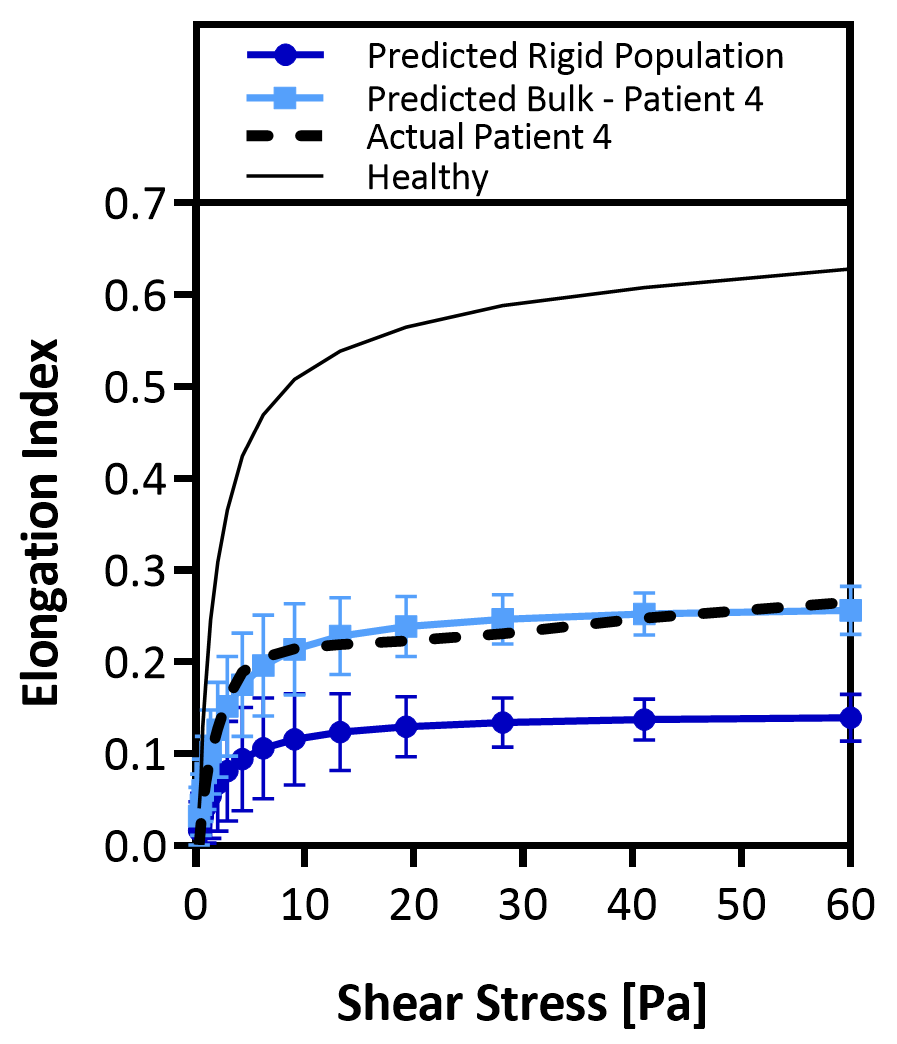


**B**


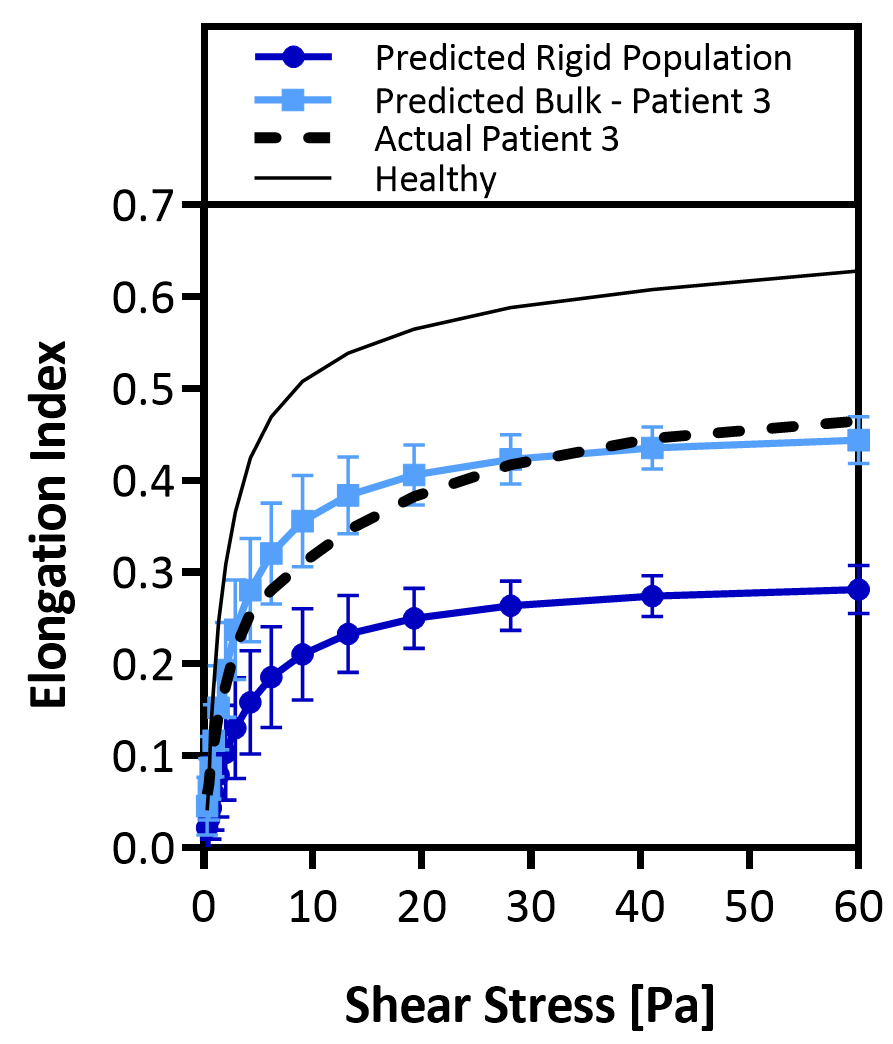

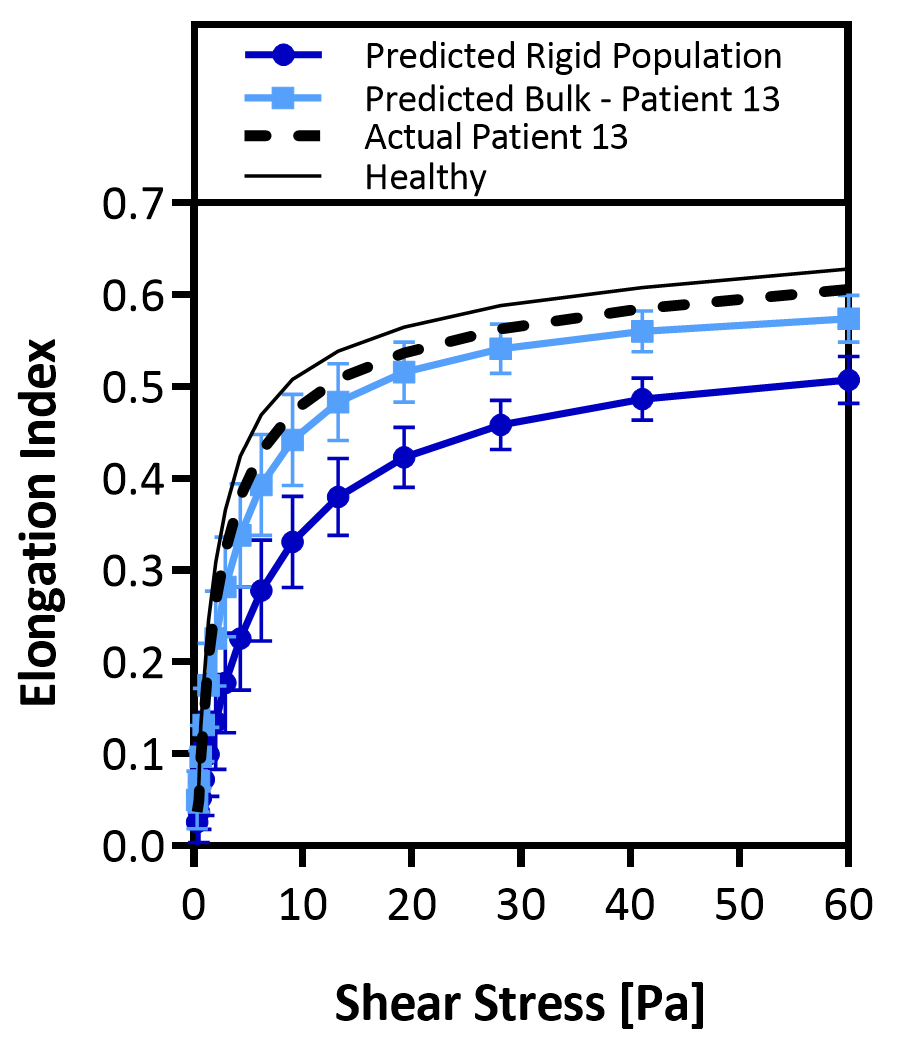


**J**


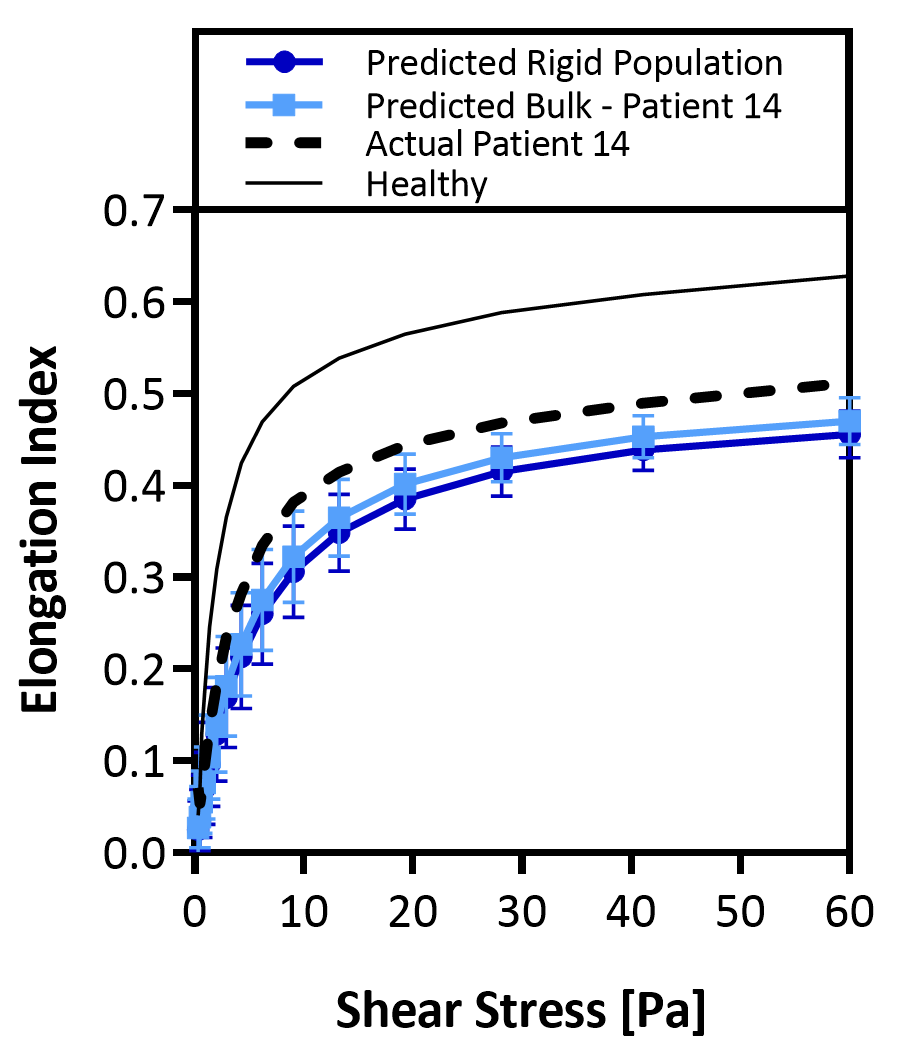


**K**


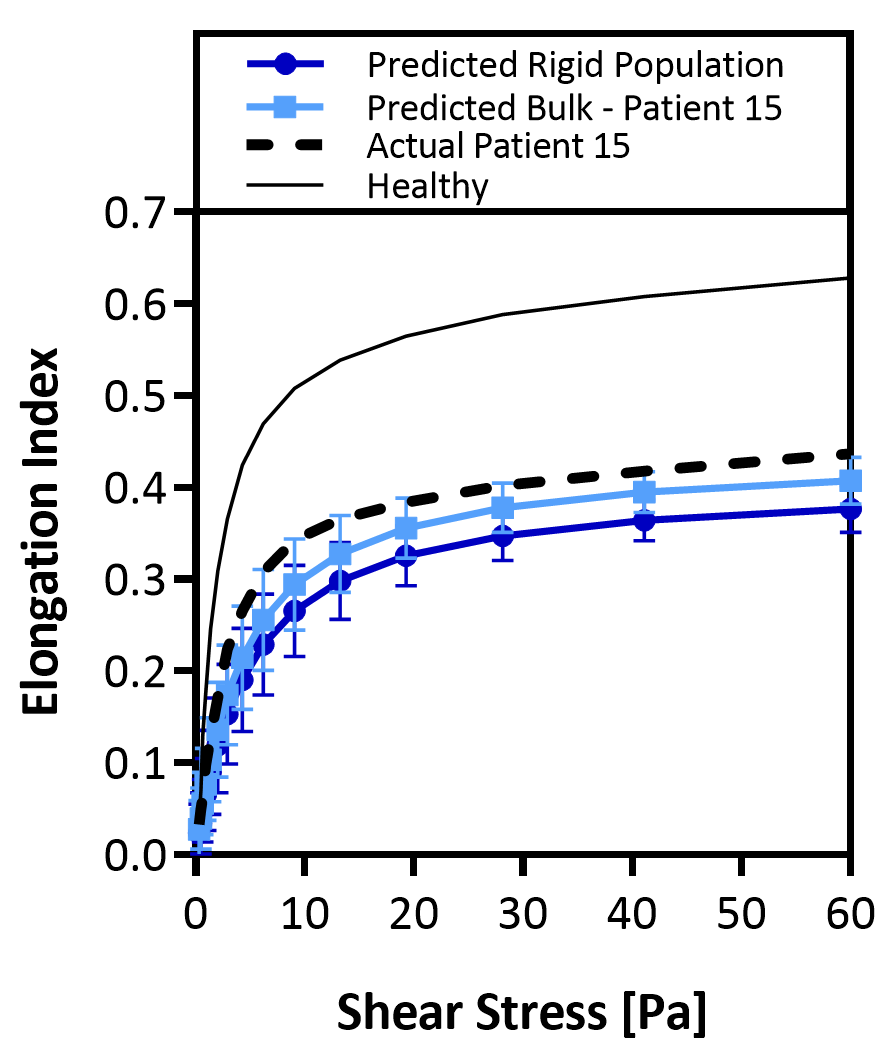


**L**
